# Long-acting injectable antipsychotics for patients with first-episode and early-phase schizophrenia: still not considered often enough

**DOI:** 10.1017/S1092852925100503

**Published:** 2025-08-19

**Authors:** Christoph U. Correll

**Affiliations:** 1 Department of Psychiatry, Northwell Health, Zucker Hillside Hospital, Glen Oaks, NY, USA; 2 Department of Psychiatry and Molecular Medicine, Donald and Barbara Zucker School of Medicine at Hofstra/Northwell, Hempstead, NY, USA; 3 Department of Child and Adolescent Psychiatry, Charité—Universitätsmedizin Berlin, Berlin, Germany; 4 German Center for Mental Health (DZPG), Partner Site Berlin, Berlin, Germany; 5 Einstein Center for Population Diversity (ECPD), Berlin, Germany

**Keywords:** Schizophrenia, first-episode, early-phase Illness, antipsychotics, long-acting injectable antipsychotics

## Abstract

Schizophrenia is a severe mental disorder with heterogeneous outcomes that depend heavily on symptom stability as a prerequisite for psychosocial rehabilitation and reintegration. Long-acting injectable antipsychotics (LAIs) are a relevant treatment tools that can help advance meaningful outcomes through improved antipsychotic adherence and relapse prevention, deliver pharmacokinetic advantages less achievable with oral formulations, improve patient autonomy, increase functioning, and reduce the risk of premature mortality even more than oral antipsychotics. However, LAIs remain largely underutilized. Non-modifiable and modifiable risk factors for relapse are summarized, potential advantages and disadvantages of LAIs are reviewed, and myths and misconceptions regarding LAIs are outlined and contrasted with evidence. This information is crucial when engaging in shared decision-making and motivational interviewing to educate patients and caregivers about the treatment option of LAIs, including in early illness stages. Since the first episode and early phases of schizophrenia are a defining time, choosing treatments with the greatest potential for improved outcomes is key. In adults with multi-episode schizophrenia, LAIs have shown superiority over oral antipsychotics for relapse/hospitalization and a variety of multiple other efficacy, effectiveness, functionality, and survival metrics. Additionally, LAIs have shown superiority over oral antipsychotics in patients with first-episode/ or early-phase illness, at least in meaningful subgroups of studies and patients that point toward superiority in settings, individuals, and treatment paradigms that more closely match clinical care. Based on this evidence, hesitancies to discuss and offer LAIs in clinical care need to be overcome, framing LAIs not as a last resort but a viable first-line/earlyphase treatment option that can meaningfully transform the long-term course of schizophrenia.

## Introduction

Schizophrenia is a heterogeneous mental disorder with both a neurodevelopmental and neurotoxic component that affects the brain and the body in multiple adverse ways.^1^ The onset of schizophrenia is often in late adolescence or early adulthood,^2^ a period that overlaps with critical stages of biological, personal, interpersonal, educational, and vocational development.

While the outcomes of people diagnosed with schizophrenia are also heterogeneous, multiple risk factors of adverse outcomes have been identified.^3^ ^,^^4^ These moderators and medicators include non-modifiable factors as well as modifiable factors that can be intervention targets. Nonmodifiable factors include male sex, earlier illness onset (especially during childhood and adolescence), premorbid developmental delay longer illness duration, and greater illness severity.^3^Modifiable factors, include a longer duration of untreated psychosis that is often substantial^5^ ^–^^7^ (necessitating early detection and intervention services),^8^ ^,^^9^ substance use comorbidity (addressed with psychosocial and/or pharmacological treatments),^10^ ^–^^13^ less early symptomatic improvement after antipsychotic initiation (indicating early informed treatment adjustments),^14^ ^,^^15^ as well as more relapses and greater non-adherence that are intricately intertwined (each being reduced by continued antipsychotic treatment, especially with long-acting injectable antipsychotics [LAIs]).^16^ ^–^^20^

Relapses that are closely related to non-adherence are particularly associated with personal, family, and societal cost, including more symptom severity and duration, more suicide attempts, less symptom improvement and more secondary treatment resistance, greater grey matter decrease than ongoing antipsychotic treatment, greater psychosocial and economic burden to patients, families, and society, as well as greater mortality risk.^21^ ^,^^22^ Since psychotic relapses play such a major role in the prediction of poorer treatment outcomes,^3^ it is also relevant to take into consideration known risk factors for psychotic relapses when designing treatment plans for and with patients living with schizophrenia. Moreover, since LAIs are a valuable tool for people with schizophrenia in general to visualize and reduce non-adherence as well as the risk of relapse and related adverse biopsychosocial downstream effects, the potential utility of LAIs from the beginning of schizophrenia and in the early illness stages should be explored. This is because people in the early illness stages are likely closest to psychosocial resources and opportunities that they can take advantage of as long as they are sufficiently symptomatically stable.

This article provides a narrative review of the role of early illness phases in schizophrenia, risk factors for psychotic relapses and their adverse downstream effects, and the potential role of LAIs, as well as counterarguments and misconceptions surrounding their use, and finally, data regarding the effectiveness and acceptability of LAIs in first-episode and early-phase schizophrenia. By challenging outdated assumptions that LAIs are only for chronically ill or nonadherent patients, the review makes the case for the earlier and broader use of LAIs as part of a recovery-oriented and patient-centered approach to the management of schizophrenia.

## Treatment goals, challenges, and results

**Figure 1.**
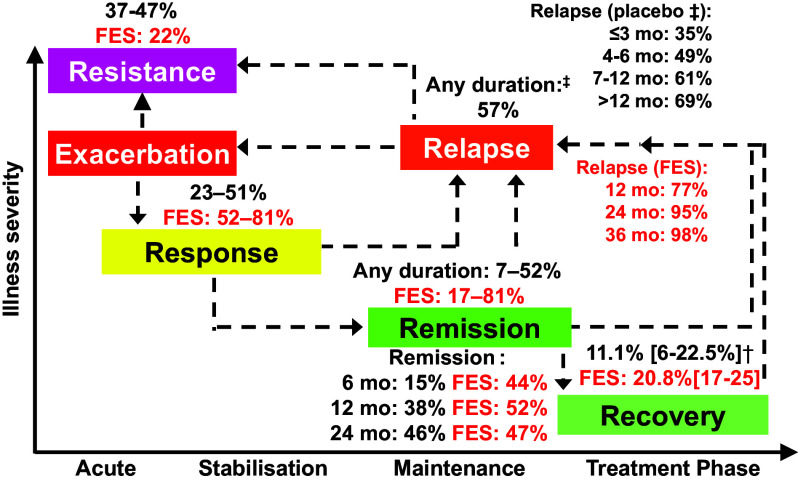
Therapeutic targets and outcomes for people with schizophrenia. †Median (interquartile range); ‡In placebo-controlled antipsychotic discontinuation studies. FES, first-episode schizophrenia; mo, month.

Figure 1 shows results from a prior review^3^ that have been updated with more recent results from meta-analyses that have quantified key outcomes in people with schizophrenia, such as antipsychotic treatment response, symptomatic remission, dyadic symptom-functioning recovery, relapse, and treatment resistance, both in first-episode (red font) and in multi-episode (black font) schizophrenia.

When patients with schizophrenia are exacerbated and acutely ill, treatment response is the first target. Research has indicated study-reported treatment response rates of 40–87% in first-episode schizophrenia and 16–65% in multi-episode schizophrenia.^3^ Since treatment response is in the eye of the beholder, quantification of that response means or is based on what is relevant. Elegant equipercentile ranking analyses comparing ratings on global psychopathology measures, such as the Positive and Negative Syndrome Scale (PANSS) or the Brief Psychiatric Rating Scale (BPRS), with changes in global illness, measured with the Clinical Global Impressions-Improvement Scale (CGI-I), have shown that a ≥ 20% reduction in PANSS or BPRS total score is equivalent to at least ‘minimally improved’ on the CGI-I, while it takes a ≥ 50% reduction in PANSS or BPRS total score to be equivalent to at least “much improved” on the CGI-I.^23^While in patients with first-episode schizophrenia, 81% and 52% were at least minimally or much/very much improved, respectively,^24^ these frequencies were much lower, 51% and 30%, respectively, in patients with multi-episode schizophrenia.^23^However, symptom response is a relative term that indicates improvement over a baseline that is variable but not wellness. Here, the concept of symptom remission is relevant. According to a widely used conceptualization, remission in schizophrenia is defined by the presence of specific and clinically relevant positive and negative symptoms of no more than mild severity.^25^The concept of remission has since been used either with the original 6-month duration criterion or cross-sectionally, according to study author’s decision. Based on study data, pooled together and using any duration criteria, remission seems more likely to occur in people with first-episode schizophrenia than multi-episode schizophrenia (17–81% vs 7–52%).^3^However, when looking more closely at the time course of symptom stability, it appears that the advantage of greater remission frequencies with first-episode schizophrenia is apparent mostly at 6 months (44% vs 15%) and 12 months (52% vs 38%), but lost at 24 months (47% vs 46%).^26^ These findings suggest that symptom worsening and relapse threaten sustained remission also in people with first-episode schizophrenia. Importantly, results for the desired outcome of recovery, i.e., the dual state of symptom stability together with functional attainment, encompassing self-care, social interactions, leisure time, and education/work, which needs to be sustained for at least 1 or even 2 years, are even lower, are even more grim. Altogether, in patients with schizophrenia across all illness stages, a median recovery rate of 13.5% has been reported without significant increases over 5 decades.^27^ When comparing patients with first-episode schizophrenia and multi-episode illness, recovery rates are higher in the early illness stage (20.8%^28^ vs 11.1%^27^) but still very low (Figure 1).

Among the reasons for the limited recovery rates, relapses score high and are among the most preventable causes.^27^In fact, when patients in double-blind randomized discontinuation trials are moved to placebo after antipsychotic stabilization, 34.8% worsen within 3 months, 48.6% within 4–6 months, 60.6% within 7–12 months and 68.4% beyond 12 months, with a pooled risk of relapse of 57.5% independent of duration of follow-up.^20^ In patients with first-episode schizophrenia, pooled weighted relapse rates after stopping antipsychotics were 77% (range: 56–91%) at 12 months, 95% (range 94–96%) at 24 months, and 98% (range (97–98%) at 36 months.^29^ Finally, relapse can lead, among other negative consequences, to decreased treatment response and even secondary treatment resistance, at least in a subgroup of vulnerable patients.^22^ Recent meta-analytic data indicate that up to 22.2% of patients with schizophrenia are antipsychotic treatment resistant from their first episode, also called primary treatment resistance, but that this rate doubles to 37–47% after multiple relapses in patients with multi-episode schizophrenia.^30^

In summary, while people with first-episode schizophrenia are more likely to initially respond to antipsychotic medications than people with multi-episode schizophrenia, these gains are lost over time. Relapses are a particular threat to maintaining symptom stability and to being able to use and translate this stability to goal attainment, including functionality and life engagement.^31^ ^–^^33^Hence, beyond acute symptom stabilization, maintenance treatment, and relapse prevention are a main building blocks to achieve desired and sustainable outcomes in people living with schizophrenia. Such sustained maintenance treatment and symptom control should start and be achieved as early as possible in the illness course.

## Knowledge about risk factors for schizophrenia relapse to inform treatment selection

Since continued antipsychotic treatment, especially with LAIs, has been shown to significantly reduce relapses in people with schizophrenia,^18^ ^,^^34^ ^,^^35^ one approach to selecting patients who may be appropriate for LAI initiation and maintenance treatment is to look for risk factors for relapse in patients with schizophrenia. Table 1 summarizes non-modifiable and modifiable risk factors for relapse, focusing on patient-related, family-related, Illness-related, and treatment-related risk factors. Results are generally derived from patients with multi-episode schizophrenia. Whenever data for risk factors for a psychotic relapse or breakthrough were available specifically for patients with first episode psychosis or schizophrenia, these results are indicted with an asterisk. Data for patients treated with LAIs are indicated by the letter “b”.

**Table 1.** Non-modifiable and Modifiable Risk Factors for Relapse in People with Schizophrenia

Patient-related	Family-related	Illness-related	Treatment-related
Non-modifiable risk factors for relapse			
Female sex: ^a^OR: 2.44 (95%CI: 1.14–5.24)^36^ Male sex: ^b^HR: 1.19 (95%CI: 1.03–1.39)^40^	Advanced paternal age: ^a^OR: 1.05 (95%CI: 1.01–1.10)^36^	Illness duration ≤5 years: 1.56 (95%CI: 1.50–1.62)^41^	More prior antipsychotic trials: OR: 1.13 (95%CI: 1.03–1.24)^45^
Younger age at illness onset: ^b^HR: 1.03 (95%CI: 1.01–1.04)^40^		^b^Greater global illness severity: HR: 1.28 (95%CI: 1.12–1.48)^40^	
Younger age: ^b^HR: 1.01 (95%CI: 1.00–1.02)^40^ Age ≤ 30 years: HR: 2.01 (95%CI: 1.88–2.14)^41^ ^b^HR: 1.87 (95%CI: 1.67–2.09)^41^		^b^Higher positive symptoms: HR: 1.04 (95%CI: 1.02–1.06)^40^	
Poorer Premorbid adjustment: OR: 2.25 (95%CI: 1.37–3.69)^42^		^b^Lower functioning: HR: 1.01 (95%CI: 1.01–1.02)^40^	
Stressful life events (1 month before relapse): OR: 2.11 (95%CI: 1.20–3.72)^43^ ^.^		Symptom improvement (vs. ‘recovery’): OR: 1.78 (95%CI: 1.34–2.38)^44^	
Modifiable risk factors for relapse			
AP non-adherence: ^a^OR: 2.37 (95%CI: 1.12–4.99)^36^ ^a^OR 4.09 (95% CI: 2.55–6.56)^42^ OR: 4.23 (95%CI: 3.32–5.38)^44^ ^a^HR: 4.8 (95%CI: 2.9–7.7)^46^ OR: 5.52 (95%CI: 2.08–14.62)^43^ AP non-adherence due to lack of illness insight: OR: 5.29 (95%CI: 2.28–12.20)^47^	High expressed emotion: ^a^Critical comments: OR: 2.35 (95%CI: 1.16–4.77)^42^	More prior hospitalizations: OR: 1.29 (95%CI: 1.21–1.36)^45^ >6 prior hospitalizations: HR: 2.40 (95%CI: 2.30–2.50)^41^ ^b^HR: 2.38 (95%CI: 2.21–2.57)^41^	AP non-adherence due to side effects: OR: 3.0 (95%CI: 1.17–7.87)^47^
Unemployment: OR: 3.04, 95%CI: 2.29–4.04)^44^ Lower income: OR: 1.83 (95%CI: 1.43–2.36)^44^	High expressed emotion: emotional over-involvement OR: 1.20 (1.09–1.36)^49^	Substance use comorbidity: ^a^OR: 2.27 (95%CI: 1.37–3.76)^42^ ^b^HR: 1.55 (1.15–2.10)^40^ Cannabis use: OR: 1.39 (95%CI: 1.12–1.72)^50^ ^a,b^OR: 4.5 (95%CI:1.4–14.6)^48^ Nicotine Smoking: ^b^HR: 1.20 (95%CI: 1.02–1.40)^40^	AP discontinuation: RR: 2.70 (95%CI: 2.33–3.13, NNH: 3.17)^19^ Intermittent AP treatment: OR: 3.36 (95%CI: 2.36–5.45) – OR: 5.64 (95%CI:4.47–7.11)^52^
Daily living difficulties: OR: 3.00 (95%CI: 2.13–4.21)^44^	Poorer communication: OR: 1.49, (95%CI: 1.02–2.17)^44^	Comorbid depressive disorder: OR: 10.57 (95%CI: 2.41–46.7)^49^ OR: 1.22 (95%CI: 1.05, 1.42)^51^ Co-morbid depressed mood: OR: 5.33 (95%CI: 2.32–12.22)^47^	AP dose reduction: RR: 1.81 (95%CI:1.41–2.38, NNH: 4.44)^19^ Slow AP dose reduction (by max. 67%) with aim to stop (27%): HR: 2.2 (95%CI: 1.2–4.0)^53^ Very low oral AP dose: RR: 1.35 (95%CI: 1.08–1.69)^54^ Very low LAI dose: RR: 1.94 (1.25–3.01)^53^
Lack of social relationships: OR: 2.61 (95%CI:1.93–3.52)^44^ Poorer social relationships: ^a,b^HR: 1.20 (95%CI:1.08–1.3)^48^		Tardive dyskinesia: ^b^HR: 2.39 (95%CI: 1.05–5.42)^40^	1st-gen. AP (vs 2nd-gen. AP): ^a^HR: 1.49 (95%CI: 1.20–1.81)^41^
Lower self-efficacy: OR: 1.25 (95%CI: 1.20–1.28)^4^			Antidepressant use: ^b^HR: 1.29 (95%CI: 1.19–1.39)^41^
			Hypnotic use: OR: 3.29 (95%CI: 2.85, 3.79)^51^ Benzodiazepine use: HR: 1.12 (95%CI: 1.08–1.17)^41^ ^b^HR: 1.31 (95%CI: 1.23–1.40)^41^ Z-drug use: HR: 1.14 (95%CI: 1.07–1.22)^41^

Abbreviations: AP, antipsychotic; HR, hazard ratio; LAI, long-acting injectable antipsychotic, OR, odds ratio; RR, risk ratio.

a Data in patients with first-episode psychosis.

b Data in patients on LAI treatment.

## Non-modifiable risk factors for relapse in schizophrenia

### Patient-related factors

Sex-based differences can influence relapse risk. Female patients with first-episode psychosis exhibit a significantly higher risk of relapse than males, with an odds ratio (OR) of 2.44 (95%CI: 1.14–5.24).^36^ Notably, 3 recent database studies in patients with first-episode schizophrenia each indicated that females were more likely to discontinue antipsychotics.^37^ ^–^^39^ Conversely, in individuals maintained on LAIs, male sex was associated with increased relapse risk (hazard ratio [HR]: 1.19, 95%CI: 1.03–1.39),^40^ possibly indicating overall greater illness severity and treatment resistance risk, despite ongoing antipsychotic treatment, which may also be related to greater risk of substance use, which is also a risk factor for relapse and breakthrough psychosis (see below).

Among LAI-treated individuals, younger age at illness onset was also associated with higher relapse risk (HR: 1.03, 95%CI: 1.01–1.04),^40^ as was younger current age, whether as a continuous risk factor (HR: 1.01, 95%CI: 1.00–1.02) or pertaining to individuals ≤30 old (HR: 1.87, 95%CI: 1.67–2.09), which was also the age group at increased risk on patients treated with oral antipsychotics (HR: 2.01, 95%CI: 1.88–2.14).^41^ Finally, poorer premorbid adjustment level further significantly raised the odds of relapse (OR: 2.25, 95% CI: 1.37–3.69).^42^Finally, stressful life events 1 month before relapse was significant related to relapse (OR: 2.11, 95%CI: 1.20–3.72).^43^

### Family-related factors

Advanced paternal age at conception is a family-related non-modifiable factor. In first-episode psychosis, each additional year of paternal age increased the odds of relapse by 1.05 (95% CI: 1.01–1.10).^36^

### Illness-related factors

Among illness-related factors, illness duration ≤5 years was related to increased relapse risk (HR: 1.56, 95%CI: 1.50–1.62).^41^ Furthermore, among LAI-treated patients,^40^ greater global illness severity (HR: 1.28, 95%CI: 1.12–1.48), higher positive symptom severity (HR: 1.04, 95%CI: 1.02–1.06) [12], and lower overall functioning (HR: 1.01, 95%CI: 1.01–1.02) were each associated with relapse risk. Finally, suboptimal improvement, defined as symptom improvement without full recovery, was also a relapse predictor (OR: 1.78, 95%CI: 1.34–2.38).^44^

### Treatment-related factors

Among treatment-related factors, the number of prior antipsychotic trials, a proxy measure for illness chronicity and insufficient response, predicted higher relapse risk (OR: 1.13, 95%CI: 1.03–1.24).^45^

## Modifiable risk factors for relapse in schizophrenia

### Patient-related factors

Among patient-related risk factors for relapse antipsychotic non-adherence is one of the most powerful modifiable predictors. Its impact is consistently large across multiple studies, with HRs and ORs ranging from 2.37 to 5.52.^36^ ^,^^42^ ^–^^44^ ^,^^46^Notably, the risk of relapse was among the highest when non-adherence was related to poor insight (OR: 5.29, 95%CI: 2.28–12.20).^47^

Further risk factors for relapse include social determinants of health, such as unemployment (OR: 3.04, 95%CI: 2.29–4.04),^44^ lower income (OR: 1.83, 95%CI: 1.43–2.36),^44^ daily living difficulties (OR: 3.00, 95%CI: 2.13–4.21),^44^ lack of social relationships (OR: 2.61, 95%CI:1.93–3.52)^44^as well as in patients with first episode schizophrenia, treated with LAIs, poorer social relationships (HR: 1.20, 95%CI:1.08–1.35).^48^Finally, lower self-efficacy was also related to relapse risk (OR: 1.25, 95%CI: 1.20–1.28).^4^

### Family-related factors

Among family-related factors, high expressed emotion, specifically critical comments in people with first-episode schizophrenia (OR: 2.35, 95%CI: 1.16–4.77)^42^ but also in general emotional over-involvement (OR: 1.20, 95%CI: 1.09–1.36)^49^ and poorer communication (OR: 1.49, (95%CI: 1.02–2.17)^44^were modifiable predictors of relapse.

### Illness-related factors

Among illness-related factors, a greater number of hospitalizations overall predicted relapse risk (OR: 1.29, 95%CI: 1.21–1.36),^45^ as did >6 hospitalizations in patients treated with oral antipsychotics (HR: 2.40, 95%CI: 2.30–2.50)^41^and similarly so when treated with LAIs (HR: 2.38, 95%CI: 2.21–2.57).^41^

A consistent and highly replicated and modifiable risk factor for relapse is substance use. Comorbid substance use increased relapse risk in people with first-episode schizophrenia (OR: 2.27, 95%CI: 1.37–3.76)^42^ as well as patients with multi-episode schizophrenia treated with LAIs (HR: 1.55, 95%CI: 1.15–2.10).^40^ Among substances, cannabis use increased the risk of relapse in general (OR: 1.39, 95%CI: 1.12–1.72),^50^whereas cannabis uses was particularly strongly implicated in relapse in patients with first-episode schizophrenia and treated with LAIs (OR: 4.5, 95%CI: 1.4–14.6).^48^ In studies where substance use was exclusionary, nicotine smoking, possible as a proxy for risk for abuse of other substances, also increased release risk modestly, even in patients receiving LAIs (HR: 1.20, 95%CI: 1.02–1.40).^40^

Furthermore, comorbid depressive disorder or depressive symptoms also increase the odds of relapse by between OR: 1.22 (95%CI: 1.05, 1.42)^51^ to OR: 5.33 (95%CI: 2.32–12.22)^47^and even OR: 10.57 (95%CI: 2.41–46.7).^49^The wide confidence intervals suggest the likely presence of subgroups and additional factors that may be related to depression and that may increase relapse further, including self-medicating substance use behaviors.

Finally, tardive dyskinesia, a potential marker of a dysregulated postsynaptic dopamine receptor system, was also associated with higher relapse risk even in patients on assured LAI treatment (HR: 2.39, 95%CI: 1.05–5.42).^40^

### Treatment-related factors

Several modifiable medication-related characteristics, behaviors, and choices influence relapse. As seen among patient-related factors, non-adherence is a crucial risk factor for relapse, and non-adherence can also be related to treatment effects, i.e., when patients decide to stop antipsychotics due to intolerable or unacceptable side effects (OR: 3.0, 95%CI: 1.17–7.87).^47^

Additional medication-related factors include antipsychotic discontinuation (RR: 2.70, 95%CI: 2.33–3.13)^19^ and intermittent treatment with the risk of relapse ranging from OR: 3.36 (95%CI: 2.36–5.45) to OR: 5.64 (95%CI:4.47–7.11), depending on duration of follow-up.^52^ But even antipsychotic dose carries an increased relapse risk (RR: 1.81, 95%CI: 1.41–2.38).^19^ This increased relapse risk also extends to slow antipsychotic dose reduction to avoid potential rebound phenomena (achieved by a maximum of 67%) with aim to stop antipsychotics (achieved in only 27%), translating into an HR for relapse of 2.2 (95%CI: 1.2–4.0).^53^ Additionally, very low oral antipsychotic doses (RR: 1.35, 95%CI: 1.08–1.69)^54^or LAI doses (RR: 1.94, 95%CI: 1.25–3.01)^53^also conferred higher relapse risk.

Among medications, first-generation antipsychotic use increased relapse risk compared to second-generation antipsychotics in people with first-episode schizophrenia (HR: 1.49, 95%CI: 1.20–1.81)^41^who are more sensitive to postsynaptic dopamine blockade.

Additional risks have been associated with adjunct psychotropic medications, possibly being markers of greater illness severity or complexity, more comorbid conditions, or difficulties with medication adherence due to too many medications. Relapse risk was increased in the presence of cotreatment with antidepressants in people on LAIs (HR: 1.29, 95%CI: 1.19–1.39),^41^benzodiazepines in people on oral antipsychotics (HR: 1.12, 95%CI: 1.08–1.17)^41^ and those on LAIs (RR: 1.31, 95%CI: 1.23–1.40),^41^ as well as with Z-drugs (HR: 1.14, 95%CI: 1.07–1.22)^41^ and especially hypnotics in general (OR: 3.29, 95%CI: 2.85–3.79).^41^

## Why LAIs make sense early in the course of illness

The notion that LAIs are only appropriate after repeated nonadherence or chronic illness is outdated and unsupported by evidence. LAIs provide multiple pharmacokinetic and clinical advantages^18^ ^,^^55^ ^,^^34^ ^,^^35^ that are especially beneficial during the vulnerable early phase of schizophrenia. LAIs bypass first-pass metabolism, minimize plasma level fluctuations with lower antipsychotic peak levels that are relevant for adverse effects in more side effect-vulnerable patients with first-episode and early-phase schizophrenia,^37^ ^–^^39^ reduce the likelihood of undetected nonadherence, which is particularly high in early phase illness, and ensure steady therapeutic exposure. These benefits translate into clinical outcomes that are both significant and relevant: lower relapse rates, fewer hospitalizations, and better functional recovery.^56^

Contrary to concerns, most patients are open to using LAIs when they understand the rationale and advantages. Studies suggest that many patients with early-phase schizophrenia prefer the simplicity and reduced burden of LAIs once they are offered as a proactive, recovery-oriented option rather than a punitive one. In fact, a 2022 Delphi consensus report involving European experts emphasized that LAIs should be considered not only in cases of proven nonadherence but also proactively based on individual risk profiles and shared decision-making.^57^ The report identified multiple clinical scenarios where early use of LAIs is appropriate: poor or uncertain adherence, insufficient insight, substance use comorbidity, family history of treatment discontinuation, and psychosocial instability. Importantly, the panel concluded that offering LAIs at the beginning of treatment can normalize their use, destigmatize them, and improve therapeutic alliances.^57^

However, despite advantages of LAIs and expert panels and guidelines advocating their use in people with schizophrenia,^56^ ^–^^61^ LAIs have remained underutilized, both in patients with multi-episode but especially in first-episode schizophrenia. For example, in an observational study using the IBM MarketScan Commercial and Medicare Supplemental databases from January 1, 2012 to December 31, 2019 including 41 391 patients with newly diagnosed schizophrenia, only 1836 (4%) received at least one LAI.^62^Moreover, only 202 (<1%) patients remained on the LAI for ≥90 days, coined “successful” LAI implementation. Notably, before LAI initiation, 58% of these patients had received ≥2 OAPs.^62^ Similarly, in a Canadian retrospective, longitudinal cohort study using pharmacy prescription data between August 2005 and June 2017, among 16 300 patients with schizophrenia-spectrum disorder, 1062 (6.5%) patients used an LAI during the 12-month study period.^63^ Of those patients, 789 used an LAI within 2 years of diagnosis (74.3% of LAI users; 4.8% of all patients). Furthermore, 65.0% of patients had been prescribed ≥2 OAPs prior to LAI use. In a UK study of 2309 patients with first-episode schizophrenia-spectrum disorder treated in South London, only 7 patients (0.3%) initiated an LAI as the first-line treatment. This number increased to 11.3% (n = 795) among 7013 treatment episodes within the first 2–5 years of treatment.^39^ These numbers can be compared to LAI use in 15% of predominantly multi-episode patients across 7 European countries based on 2011 IMS Institute for Health Care Informatics data, ranging from 7% in Switzerland to 22% in the UK.^64^

## The expanding role of long-acting injectable antipsychotics in schizophrenia

The global landscape of schizophrenia treatment is undergoing a slow but meaningful transformation, driven in part by the growing development and integration of LAIs into the treatment paradigm. While the uptake of LAIs remains uneven and still falls short of their full clinical potential, LAIs are increasingly recognized not merely as tools to reduce nonadherence, but as proactive agents of stabilization, relapse prevention, and long-term functional recovery. Across diverse health systems, there is growing awareness of LAI benefits in preventing hospitalization, improving quality of life, and even extending life expectancy, which is significantly reduced in schizophrenia, by ensuring consistent therapeutic coverage.^65^ ^–^^70^

This shift reflects an evolution in both evidence and mindset toward more proactive rather than reactive care and toward early interventions aiming to provide relevant benefits at critical illness episodes.^9^ The clinical utility and versatility of LAIs has recently expanded through increasing pharmacokinetic precision that have enabled the development of longer-acting LAIs, with more 2-monthly, 3-monthly and, even 6-monthly LAI formulations to choose from.^71^ ^–^^76^ Furthermore, subcutaneous LAIs have emerged that provide different needle size and length and injection site options^71^ ^,^^74^ and may avoid adverse effects associated with deep intramuscular injections, such as the post-injection delirium somnolence syndrome that can occur in approximately every 1200 injections with intramuscular injectable olanzapine pamoate.^74^ Moreover, LAI formulations have become available without the need for oral supplementation, loading doses or booster injections.^71^ ^,^^74^ ^,^^77^ Clinical trials and real-world data now also support earlier and broader use of LAIs, marking a departure from their historical positioning as a last resort for nonadherent or severely ill patients.^78^ ^–^^82^ In tandem, these agents are being integrated into shared decision-making frameworks and supported by digital adherence tools, empowering clinicians and patients alike to view LAIs as cornerstones of sustained recovery, not just crisis management.^83^

Despite this progress, however, substantial barriers remain. Unequal access, lingering stigma, gaps in clinician training—especially around motivational interviewing and psychoeducation—continue to limit LAI adoption. Yet, where LAIs are most rapidly embraced, such as in early intervention services and assertive community treatment, their transformative potential is being realized.^84^ Still, a key hurdle to broader implementation is persistent misinformation. Outdated assumptions and entrenched myths continue to shape clinician and patient attitudes, hindering the full integration of LAIs into routine care.

## Pros and cons of long-acting injectable antipsychotics (LAIs)

Although compared to oral antipsychotics, LAIs offer a number of clinical, functional, and economic advantages, their adoption remains uneven. A clear understanding of both the benefits and drawbacks of LAIs is essential for informed shared decision-making between clinicians, patients, and caregivers. Knowledgeable clinicians who can effectively and compassionately present information are particularly important when treating patients in the early illness phases of schizophrenia. At these illness stages, patients and caregivers often still grapple with the shock of the illness, accepting the diagnosis and required treatment. Also, patients (and caregivers) may be distrustful, and only partially informed about the illness and treatment options. Table 2 summarizes main potential advantages and disadvantages of LAIs require consideration when offering LAIs as part of a comprehensive treatment plant.^18^ ^,^^34^ ^,^^35^ ^,^^65^ ^,^^67^ ^–^^70^ ^,^^85^ ^–^^87^

**Table 2.** Potential Advantages and Disadvantages of Long-Acting Injectable Antipsychotics (LAIs)

Potential advantages of LAIs	Potential disadvantages of LAIs
Greater medication persistence and adherence	Injection site pain and local reactions
Sustained symptom control and relapse prevention	Anxiety or distress related to needle use
Reduced risk of hospitalization and emergency visits	Limited antipsychotic choices available as LAIs
Immediate awareness of non-adherence when injections are missed	Slower dose titration
Better differentiation between treatment failure and non-adherence	Slower discontinuation in the event of side effects or patient preference
Identification of true vs. pseudo-treatment resistance	Risk of hematomas in patients on blood thinners (IM only)
Clinical time can be focused on goals and recovery rather than adherence	More time needed to educate patients on LAI procedures
Stable plasma drug levels with reduced peak–trough fluctuations	Persistent stigma due to past use in treatment-resistant or coercive settings
Lower total medication dose due to pharmacokinetic efficiency	Requires infrastructure for administration and monitoring
Elimination of overdose risk	Higher drug acquisition cost (though cost-effective overall)
Reduced peak-related side effects (e.g., hyperprolactinemia)	
Improved functioning, cognition, and vocational engagement	
Greater patient and caregiver satisfaction	
Lower direct and indirect healthcare costs	
Reduced all-cause mortality, including suicide-related and cardiovascular disease-related deaths	

## Advantages of LAIs

One of the most significant benefits of LAIs lies in their ability to ensure sustained medication adherence. Because LAIs are administered by healthcare providers at regular intervals (e.g., biweekly, monthly, bimonthly, quarterly, even 6-monthly), they effectively eliminate the variability and uncertainty of daily oral medication intake. This approach allows for greater treatment persistence, more reliable symptom control, and more robust prevention of relapses and hospitalizations. LAIs also facilitate earlier identification of non-adherence—when a patient misses an injection appointment, this can be recognized immediately, unlike missed oral doses, which often go unnoticed, and appropriate action can be taken to understand the patient’s reasons for discontinuation and to take the best next steps.

From a diagnostic standpoint, LAIs help differentiate between true treatment failure and non-response due to poor adherence, substance use, or psychosocial stressors. LAIs help clarify whether a patient has true pharmacologic treatment resistance or “pseudo-resistance” caused by inconsistent oral dosing. This distinction is crucial for optimizing treatment decisions and avoiding unnecessary medication changes.

Pharmacokinetically, LAIs maintain more stable plasma levels, avoiding the more pronounced peaks and troughs associated with daily oral antipsychotics. This stability may lead to improved tolerability, fewer breakthrough symptoms, and reduced side effects linked to peak concentrations, such as hyperprolactinemia. Additionally, the total drug exposure may be lower, as LAIs often require lower cumulative doses while maintaining therapeutic levels. By way of comparison, 400 mg aripiprazole-LAI is equivalent to about 20 mg oral aripiprazole, so that 20 mg x 30 days would yield 600 mg aripiprazole-LAI but only 400 mg aripiprazole-LAI are required to not fall below the minimum required blood level, sparing higher doses with greater resultant peak levels.^55^ ^,^^71^Similarly, 156 paliperidone-LAI once-monthly mg is equivalent to 9 mg oral paliperidone, so that 9 mg × 30 days would yield 270 mg paliperidone-LAI once-monthly but only 156 mg paliperidone-LAI once-monthly are required to result in equivalent maintenance through blood levels and effectiveness.^55^ ^,^^71^Furthermore, LAIs also eliminate the risk of intentional or accidental overdose.

Functionally, patients receiving LAIs tend to show improved daily functioning, better cognitive performance, and increased vocational participation. Caregivers often report reduced stress due to the predictability and visibility of adherence. Economic models suggest that although LAIs may incur higher upfront medication costs, they lead to downstream savings through reduced hospitalization, emergency care, and mortality—particularly by reducing suicide risk and deaths from cardiovascular or other natural causes.^67^ ^,^^68^

## Potential disadvantages of LAIs

Despite these compelling advantages, LAIs are not without limitations. Some patients experience injection site pain or anxiety related to needles, which can deter use. The limited number of antipsychotic compounds available in long-acting formulations restricts therapeutic flexibility, especially for patients with complex comorbidities or intolerance to specific antipsychotics.

Dose adjustments with LAIs are inherently slower due to their extended pharmacokinetics. This also complicates treatment discontinuation, particularly in cases of emergent side effects or patient preference. In individuals taking anticoagulants, intramuscular injections may lead to hematomas, although this risk does not apply to newer subcutaneous LAIs.

Implementing LAI treatment also requires additional time and infrastructure. Clinicians need to explain the administration process in detail, address patient concerns, and coordinate regular appointments. Stigma remains another barrier: the historical association of LAIs with coercive or last-resort treatment still affects patient and provider attitudes. Finally, while LAIs tend to reduce overall healthcare costs, the medication itself may be more expensive than oral formulations, presenting access challenges in some settings.

## Dispelling myths about long-acting injectable antipsychotics: elevating evidence over assumptions

Despite robust evidence, several misconceptions continue to impede the widespread adoption and also the early use of LAIs in patients with schizophrenia. Dispelling the most common misconceptions is critical for increasing the appropriate and timely use of LAIs. Many of these myths stem from outdated frameworks or anecdotal impressions that equate LAI use with coercion, chronicity, or therapeutic inflexibility. These misconceptions can lead to suboptimal treatment choices. A closer look at the facts can help dispel these myths and underscores the evolving role of LAIs in modern psychiatric care.

Table 3 summarizes 10 of the most pervasive myths about LAIs, as well as factual information that can be shared when patients or caregivers voice such misconceptions.^85^ Additionally, one may even raise and address those objections proactively, as patients and caregivers may not feel comfortable bringing them up themselves, or as they may be confronted with misinformation after the clinical visit via conversations or the internet.

**Table 3.** 12 Misconceptions and Facts about Long-Acting Injectable Antipsychotics

Misconception	Fact
1. LAIs are only for nonadherent or chronic patients	Early LAI use improves outcomes in first-episode and early-phase psychosis, reducing relapse, functional decline and disability and early mortality, especially due to suicide.
2. LAIs reduce autonomy or are coercive	When framed through a shared decision-making paradigm, LAIs enhance autonomy by reducing medication burden and relapse risk, enhancing goal attainment opportunities.
3. Patients will not accept LAIs	When offered early and appropriately, many patients prefer LAIs for their convenience and reliability and when framed in a motivational interviewing style, for their ability to help patients better achieve their own goals.
4. LAIs only improve adherence	LAIs provide pharmacokinetic benefits—fewer relapses, hospitalizations, and certain antipsychotic peak level-dependent side effects—even in adherent patients.
5. LAIs cause more side effects than oral antipsychotics	Meta-analyses show comparable or better tolerability, especially through reduced peak plasma levels.
6. LAIs are associated with a higher risk of neuroleptic malignant syndrome or poorer health outcomes	Although after LAI discontinuation blood levels remain in the system for a longer time than with OAPs, multiple different observational data sources indicate that the risk for NMS or for related adverse health sequelae is not increased with LAIs.
7. LAIs should be a “last resort” option	Delaying LAI use until oral antipsychotic failure denies patients early stabilization benefits.
8. LAIs are too inflexible	While slower to adjust, LAIs offer greater predictability and long-term stability, helping also to clarify side medication related side effects or lack of efficacy vs. medication unrelated physical or mental symptoms, including pseudo-treatment resistance
9. LAIs increase stigma	The association between LAIs and stigma reflects clinician bias more than patient perception or reality. When presented as standard care, especially in early illness, stigma diminishes. Similarly, when stability and functional outcomes increase, stigma also diminishes.
10. Offering LAIs takes too much time	Offering LAIs can be done over multiple visits and be supported by handouts, websites and/or patient videos. The improved stability and outcomes ultimately save time.
11. Prescribing LAIs is too costly	The improved stability and outcomes associated with LAIs ultimately save money and resources.
12. LAIs, shrink the brain and are neurotoxic.	Patients on vs. off antipsychotics have somewhat reduced brain volumes in structural imaging studies, but brain volumes reduce 3 times more with relapses and ongoing psychosis, brain areas of patients on antipsychotics are better connected, and cognition is improved compared to not being on antipsychotics; thus, LAIs may reduce neurotoxic progression even better than oral antipsychotics.

Abbreviations: LAI, Long-acting injectable antipsychotic; NMS, neuroleptic malignant syndrome; OAP, oral antipsychotic.

A common myth is that LAIs are reserved solely for patients who are nonadherent or chronically ill. However, robust data now demonstrate that early use of LAIs, particularly in first-episode and early-phase psychosis, yields superior outcomes.^78^ ^–^^82^ These benefits include reduced relapse rates, delayed illness progression, and improved functional recovery. Rather than being a last resort, LAIs should be considered early in the course of illness to maximize long-term benefits.

Another frequent concern is that LAIs reduce patient autonomy or feel coercive. In contrast, when introduced within a shared decision-making framework, LAIs can enhance autonomy. By minimizing the daily burden of pill-taking and protecting against destabilizing relapses, patients often experience greater freedom to pursue life goals, employment, and meaningful relationships without the fear of relapse and the adverse life consequences related to clinical deterioration.^88^

It is also often assumed that patients will resist and not accept LAIs. Yet studies and clinical experience consistently show that when LAIs are presented early, without bias, and with a clear explanation of their benefits, many patients find them preferable due to their convenience, discretion, and reliability. Rather than rejecting LAIs, a significant proportion of patients value the reduced treatment burden and enhanced sense of security they provide.^88^ ^–^^90^

Some clinicians believe that LAIs benefit only those who are poorly adherent, but this overlooks their pharmacokinetic advantages.^55^ Even in patients who reliably take oral medications, LAIs reduce fluctuations in drug levels, lower the risk of breakthrough symptoms, and diminish the likelihood of hospitalization. These benefits are independent of adherence and stem from more stable and sustained plasma concentrations.

Concerns about increased side effects are also misplaced. Meta-analyses and head-to-head comparisons show that LAIs are generally as well tolerated as oral formulations—and in some cases better tolerated—due to the minimization of high plasma peaks that can trigger adverse effects like akathisia or hyperprolactinemia.^86^

Relatedly, there have been concerns that LAIs are associated with a higher risk of neuroleptic malignant syndrome (NMS) or poorer related health outcomes. If it were true, this argument against safe LAI use would be particularly relevant for longer-injection interval LAIs, as in addition to symptomatic management of the severe muscle stiffness and hydration to minimize the risk of massive myoglobin breakdown and resultant acute renal failure, cessation of the offending agent is generally recommended. However, although after LAI discontinuation blood levels remain in the system for a much longer time than with OAPs, a Finish nationwide observational data,^91^ a comprehensive analysis of case reports,^92^ ^,^^93^ and a Japanese spontaneous adverse event reporting database study^94^ indicate that the risk for NMS or for related adverse health sequelae is fortunately not increased with LAIs.

A particularly harmful myth is that LAIs should only be used after oral antipsychotic treatment fails. This belief delays timely stabilization and increases the risk of relapse, hospitalization, and functional decline. Early introduction of LAIs is associated with fewer relapses and better long-term outcomes, challenging the idea that LAIs should be a fallback strategy.^78^ ^–^^82^ ^,^^84^

While it is true that LAIs have slower dose adjustment profiles, this is not inherently a drawback. Rather, the pharmacologic predictability and consistency of LAIs can help clarify whether clinical deterioration stems from insufficient dosing, underlying illness progression, or emerging side effects, insights that can be obscured by erratic oral dosing patterns.^34^ ^,^^95^

Another barrier is the stigma associated with LAIs, often linked to the misperception that injectable treatment implies a severe or “difficult” diagnosis. However, stigma is not inherent to LAIs, it is shaped by how clinicians frame the discussion. When LAIs are normalized and offered as a modern, evidence-based treatment option, patients are more likely to accept them and appreciate their benefits.^89^ ^,^^90^ ^,^^96^ ^,^^97^

There is also a belief that LAIs are too time-consuming or costly to implement. While initiating LAIs may require upfront effort, including education, scheduling, and infrastructure, the long-term return is substantial. LAIs prevent costly crises, reduce emergency visits and rehospitalizations, and enable more productive clinical encounters focused on recovery and rehabilitation rather than damage control.^36^ ^,^^98^ ^–^^100^ Moreover, more widespread community pharmacy delivery of the LAI injections may reduce structural barriers to LAI use in the future.^101^

Finally, some critics raise neurotoxicity concerns, claiming that long-term antipsychotic use, especially in LAI form, shrinks the brain. This worry may deter especially patients with early-phase illness and their caregivers from considering LAI treatment options. However, research has clarified that relapse, not antipsychotic medication, is the major driver of brain volume loss in schizophrenia that is functionally relevant.^102^ ^–^^105^ LAIs, by preventing relapses more effectively than oral agents, may offer a protective effect against neuroprogression and support the preservation of cognitive function.

In sum, outdated myths about LAIs hinder their appropriate use and do a disservice to patients. As the evidence base continues to grow, it is incumbent upon clinicians to shift from assumption to science, and to engage in transparent, nonjudgmental discussions with patients about the full range of treatment options available.

## Relevance of first-episode and early-phase schizophrenia

The first-episode and early phase of schizophrenia represents a defining moment in a person’s illness trajectory. In the initial phase of illness, patients are particularly vulnerable due to limited insight, denial of illness, or struggles to accept the illness, insufficient or slow symptom improvement in some and adverse effects of the antipsychotics in other cases, cognitive deficits, and often severe psychosocial disruption. Many patients lack awareness of the need for treatment and are therefore at high risk of medication nonadherence.^80^ ^,^^106^ The consequences of early treatment discontinuation are highly predictable, even though schizophrenia can have a varied course: symptom exacerbation, neurobiological decline, increased hospitalizations, suicide risk, and worsening psychosocial outcomes,^22^ ^,^^107^ results that are especially devastating when patients are trying to get back on their feet after a first psychotic episode.

On the other side, the early illness phase also offers the greatest chance to improve long-term prognosis. Studies show that comprehensive early intervention programs that include pharmacological treatment, psychoeducation, family support, and vocational rehabilitation are associated with better symptom control, greater adherence, and improved quality of life.^9^ Within this framework, ensuring pharmacologic continuity is essential, and LAIs can play a transformative role for patients in their first episode and early illness phases of schizophrenia.^108^ ^,^^109^In fact, a 3-year, longitudinal, prospective, naturalistic study of 416 patients with FEPs admitted to early intervention services in Canada showed that LAIs were able to “rescue” patients with poor baseline prognostic factors when they were started on LAIs instead of OAPs as early maintenance treatment.^110^ In those patients most vulnerable for interruptions of active psychosocial reintegration efforts, psychotic relapse rates over time were similar to those patients with FEP and good baseline prognostic factors who only received OAPs. In contrast, patients who initially received OAPs and only eventually switched to LAIs were more likely to relapse and to be rehospitalized, even if they manifested better baseline prognostic factors than those started initially on LAIs.

## LAIs versus oral antipsychotics in first-episode and early-phase schizophrenia

The currently most comprehensive meta-analysis of LAIs in early-phase schizophrenia pooled data from 11 randomized controlled trials comparing LAIs head-to-head against oral antipsychotics in 2374 adults with first episode or early-phase schizophrenia.^109^ Patients were on average 25.2 years old, 68% were males, and the median illness duration was 10.6 months. Relapse or hospitalization and all-cause discontinuation (“acceptability”) at study-endpoint were the co-primary outcomes. Prespecified subgroup analyses aimed to identify factors moderating differences in efficacy or acceptability between LAIs and oral antipsychotics.

Across the 11 trials with a median duration of 78 weeks (range: 13–104 weeks), LAIs were not significantly different from oral antipsychotics for the prevention of relapse or hospitalization (RR: 0.79, 95%CI: 0.58–1.06, p = 0.13) and all-cause discontinuation (RR: 0.92, 95%CI: 0.80–1.05, p = 0.20). However, results were each in the direction of favoring LAIs and the results as well as trial, patient, and treatment characteristics were each highly heterogeneous.

In addition to the heterogeneity of the results, one needs to also consider that it is much more difficult to show LAI superiority versus oral antipsychotic treatment. The difficulty regarding differentiation from oral antipsychotics regarding the effectiveness outcome can be due to (i) greater adherence in randomized controlled settings across treatment groups, (ii) randomization bias of less severely ill patients than are eligible for LAI use in usual care settings, those with greater illness insight and better cognitive capacity, (iii) surveillance bias in patients knowing that their adherence will be checked, and (iv) optimized procedures compared to usual care (e.g. reminders and incentives for visit adherence, handing out medications at the visit, etc.), which each elevate adherence and outcomes preferentially in the oral antipsychotic comparator arm.^111^ Additionally, in randomized trials comparing LAIs with oral antipsychotics, often the oral antipsychotic can be selected based on prior experiences and patient preferences, whereas usually the single tested LAI is set and cannot be chosen. Since antipsychotics differ the most in their adverse event profile, this procedure also selectively favors oral antipsychotics regarding the acceptability outcome.^111^

## Subgroup moderators of long-acting injectable antipsychotic superiority in first-episode or early-phase schizophrenia

In the meta-analysis by Vita et al.^109^ the significant heterogeneity of the results prompted the per-protocol conduct of prespecified subgroup analyses that were designed to assess significant factors that may moderate (baseline factors) or mediate (intra-treatment factors) the outcome differences between LAIs and oral antipsychotics in patients with first-episode or early-phase schizophrenia.

These subgroup analyses, summarized in Table 4, reveal that the benefits of LAIs are not uniformly distributed across all study contexts and patient populations. Several characteristics significantly moderate the comparative effectiveness of LAIs, either for relapse prevention, treatment acceptability, or both. Below, these findings are summarized by grouping moderators into 4 categories, based on which outcome(s) showed significant improvement with LAIs, and ordered by descending effect strength.

**Table 4.** Subgroup Analyses Comparing Long-Acting Antipsychotics with Oral Antipsychotics in Patients with First-Episode or Early-Phase Schizophrenia Based on Design, Patient Population, Treatment Approach and Data Analysis Features of the Randomized Trials

#	Study characteristic	Subgroup analysis	Relapse risk, RR (95%CI)	All-cause discontinuation (“Acceptability”), RR (95%CI)
Variables significantly moderating superiority of LAIs vs oral antipsychotics only for relapse prevention				
1	Analysis based on strict intent-to-treat approach	Yes	0.64 (0.52–0.80)***	0.88 (0.73–1.07)
		No	1.09 (0.72–1.65)	0.99 (0.86–1.13)
2	Pragmatic vs explanatory study characteristic (≥ or < than median ASPECT-R score of included studies)	Pragmatic studies	0.67 (0.54–0.82)***	0.94 (0.67–1.32)
		Explanatory studies	0.89 (0.47–1.68)	0.93 (0.85–1.02)
3	Oral antipsychotic selection based on previous treatment history	Yes	0.71 (0.53–0.96)*	0.99 (0.59–1.68)
		No	0.80 (0.51–1.25)	0.91 (0.82–1.01)
Variables significantly moderating superiority of LAIs vs oral antipsychotics for both relapse prevention and “acceptability”				
4	Clinically stable patients (according to mean total psychopathology scores)	Stable Patients	0.65 (0.42–0.92)*	0.99 (0.72–1.36)
		Unstable Patients	0.98 (0.62–1.56)	0.89 (0.81–0.99)*
Variables significantly moderating superiority of LAIs vs oral antipsychotics only for “acceptability”				
5	Exposure to the same oral antipsychotic ≥2 weeks Before Randomization to the LAI	Yes	0.76 (0.47–1.22)	1.00 (0.82–1.24)
		No	0.80 (0.49–1.32)	0.84 (0.72–0.98)*
6	Narrow or broad schizophrenia definition	Only Schizophrenia	0.79 (0.55–1.13)	0.87 (0.79–0.95)**
		Schizophrenia-Spectrum Disorder	0.73 (0.38–1.40)	1.08 (0.78–1.48)
7	Illness duration	Only ≤2 Years From First Episode	0.52 (0.22–1.30)	1.01 (0.61–1.67)
		Also >2 Years From First Episode	0.87 (0.61–1.24)	0.88 (0.80–0.97)**
8	Oral antipsychotic supplementation of the LAI arm allowed	Yes	0.74 (0.53–1.03)	0.90 (0.81–0.99)*
		No	1.02 (0.48–2.18)	0.99 (0.59–1.64)
Variables not significantly moderating superiority of LAIS vs oral antipsychotics for either relapse prevention or “acceptability”				
9	LAI antipsychotic class	First-generation antipsychotic	0.81 (0.15–4.45)	1.83 (0.33–10.02)
		Second-generation antipsychotic	0.78 (0.57–1.08)	0.91 (0.79–1.04)
10	Measure of adherence in the oral antipsychotic arm with a proxy measure	Yes	0.78 (0.54–1.14)	0.96 (0.82–1.13)
		No	0.81 (0.36–1.85)	0.81 (0.62–1.04)
11	High risk of bias	Yes	1.05 (0.80–1.38)	1.32 (0.72–2.41)
		No	0.40 (0.13–1.24)	0.91 (0.82–1.00
12	Pharmaceutical company funding/sponsorship	Yes	0.79 (0.58–1.09)	0.92 (0.80–1.05)
		No	0.60 (0.14–2.61)	0.91 (0.25–3.36)

Note. Based on Vita et al.^109^

Abbreviations: ASPECT-R, A Study Pragmatic-Explanatory Characterization Tool-Rating; CI, Confidence Interval; ITT, Intention-to-treat; LAI, Long-acting Injectable antipsychotics; RR, Risk Ratio.

* *p*-value <0.05. ** *p*-value <0.01. *** *p*-value <0.001.

Bold values are statistically significant at *p*<0.05.

### Variables significantly moderating superiority of LAIs only for reducing relapse risk

Three trial or treatment features significantly moderated the superiority of LAIs over OAPs exclusively in terms of reducing the risk of relapse, without a concurrent significant effect on all-cause treatment discontinuation.^109^

The first characteristic was whether the study employed a strict intention-to-treat analysis. In such trials, LAIs significantly reduced relapse risk compared to OAPs, with a risk ratio (RR) of 0.64 (95% CI: 0.52–0.80), while there was no significant difference in treatment acceptability (RR: 0.88; 95% CI: 0.73–1.07). This result suggests that rigorous analytical frameworks are more likely to capture the true clinical benefit of LAIs.

The second characteristic was the degree of pragmatism in trial design, as indexed by the ASPECT-R tool. In studies with a more pragmatic orientation, better reflecting real-world settings. Here, LAIs showed significantly better relapse prevention (RR: 0.67; 95% CI: 0.54–0.82), but no difference in acceptability (RR: 0.94; 95% CI: 0.67–1.32). This finding implies that LAIs may be particularly effective in everyday clinical contexts.

The third significant moderator in this category was whether the oral comparator was selected based on a patient’s prior treatment history. When oral antipsychotics were chosen with reference to prior exposure or response, LAIs still demonstrated a significant advantage in relapse prevention (RR: 0.71; 95% CI: 0.53–0.96), but not in all-cause discontinuation (RR: 0.99; 95% CI: 0.59–1.68), underscoring the value of LAIs, even when the oral antipsychotic choices could be tailoring.

### Variables significantly moderating superiority of LAIs for both relapse risk and acceptability

In one subgroup comparison, LAIs were found to be significantly superior to OAPs in reducing both relapse risk and all-cause discontinuation, highlighting a dual benefit.^109^

The key characteristic in this category was clinical stability, based on mean total psychopathology scores. Among patients classified as clinically stable, LAIs significantly reduced the risk of relapse (RR: 0.65; 95% CI: 0.42–0.92) versus oral antipsychotics, while no significant difference was found in acceptability (RR = 0.99; 95% CI: 0.72–1.36). In contrast, among clinically unstable patients, LAIs showed no significant reduction in relapse (RR: 0.98; 95% CI: 0.62–1.56) but did demonstrate improved acceptability (RR: 0.89; 95% CI: 0.81–0.99). Taken together, this suggests that LAIs may offer more comprehensive advantages in clinically stable individuals, arguing for a more robust oral antipsychotic treatment stabilization phase before transitioning to an LAI in people with first-episode and early-phase schizophrenia. Nevertheless, even in more unstable patients, treatment continuation is higher with LAIs than on oral antipsychotics.

### Variables significantly moderating superiority of LAIs only for acceptability

Four features were associated with a statistically significant advantage for LAIs in reducing all-cause discontinuation rates, without corresponding significance for relapse prevention.^109^

The first characteristic was prior exposure to the same oral antipsychotic for at least 2 weeks before randomization. In studies where this exposure was not present, LAIs significantly improved acceptability (RR: 0.84; 95% CI: 0.72–0.98), although relapse reduction did not reach significance (RR: 0.80; 95% CI: 0.49–1.32). Again, LAIs seem to be able to keep patients longer in treatment who are not that stable yet or who have less experience with the current antipsychotic.

The second characteristic was diagnostic groups. Trials that included only patients with a strict diagnosis of schizophrenia, excluding broader spectrum disorders, demonstrated a significant reduction in discontinuation with LAIs (RR: 0.87; 95% CI: 0.79–0.95) but no corresponding benefit in relapse risk (RR: 0.79; 95% CI: 0.55–1.13).

The third significant moderator was illness duration. In studies that also included patients with a duration of illness exceeding 2 years from first episode, LAIs showed improved acceptability (RR: 0.88; 95% CI: 0.80–0.97), while relapse risk was not significantly different (RR: 0.87; 95% CI: 0.61–1.24). This finding suggests a continuing benefit of LAIs beyond the very early phase of illness, particularly in helping patients stay on treatment.

The fourth characteristic was whether oral antipsychotic supplementation was allowed in the LAI treatment arm. In trials permitting such supplementation, LAIs showed significantly lower all-cause discontinuation (RR: 0.90; 95% CI: 0.81–0.99), while their effect on relapse remained non-significant (RR: 0.74; 95% CI: 0.53–1.03). This result points to the utility of flexible LAI protocols in enhancing acceptability and persistence.

## Variables not significantly moderating superiority of LAIs for either outcome

Finally, 4 study or treatment characteristics did not significantly moderate the comparative effectiveness of LAIs for either relapse prevention or treatment acceptability.^109^ These included whether (i) the LAI was a first-generation or second-generation antipsychotic, (ii) a proxy adherence measure was used in the oral treatment arms; (iii) studies had high or low risk of bias, and (iv) the trials were industry-sponsored or not.

Taken together, these subgroup findings demonstrate that the superiority of LAIs over oral antipsychotics in early-phase schizophrenia is not universal but is meaningfully moderated by trial design, patient characteristics, and implementation features. However, it is also possible that given the still modest number of trials and participants, subgroup analyses may have been underpowered. Since the vast majority of the 24 subgroup estimates per co-primary outcome had RRs <1 (relapse: 21/24 = 87.5 subgroup outcomes; acceptability: 19/24 = 79.2% subgroup outcomes), indicating at least numerical trends favoring LAIs estimates, future studies should be conducted to further explore overall and subgroup benefits of LAIs vs oral antipsychotics in the early stages of schizophrenia. Nevertheless, based on the available findings, relapse prevention benefits are more clearly seen in rigorous and pragmatic study designs, while improved treatment continuation emerges in broader clinical contexts, encompassing also more unstable or clinically complex cases. Recognizing these moderators can guide both clinical decision-making and the design of future trials to target or identify populations most likely to benefit from LAI formulations.

## Conclusions

The evidence summarized above indicates that LAIs can advance meaningful outcomes through improved antipsychotic adherence and relapse prevention, deliver pharmacokinetic advantages less achievable with oral formulations, improve patient autonomy, increase functioning, and reduce the risk of premature mortality even more than oral antipsychotics (except for clozapine in patients with treatment-refractory illness^68^). Additionally, LAIs have shown superiority over oral antipsychotics in patients with first-episode or early-phase illness, at least in meaningful subgroups of studies and patients that point toward evidence in settings, individuals, and treatment paradigms that more closely match clinical care.^109^

Based on this evidence, recommendations for clinical practice and policy indicate that a reevaluation of how and when LAIs are introduced in the treatment of schizophrenia is needed. Clinicians, policymakers, and healthcare systems must overcome hesitancy in adopting LAIs early. This task requires concerted educational efforts, guideline updates, and structural reforms to facilitate LAI administration across care settings. Importantly, framing LAIs not as a last resort, but as a viable first-line or early-phase option, is likely to meaningfully transform the long-term course of schizophrenia.

Clinicians should consider offering LAIs early in the illness course, especially when adherence is uncertain or risk factors for relapse are present. However, adherence is almost always uncertain and risk factors for relapse are abundant and often interrelated. Therefore, almost all patients with schizophrenia, from the first episode onwards, can and should be informed about the treatment option of LAIs, educating them proactively about the potential pros and cons of LAI treatment options. This approach aligns with emerging consensus and expert guidelines, which support earlier use of LAIs in appropriate patients.^56^ ^–^^61^

Training programs for all providers of care to patients with schizophrenia, including, psychiatrists, psychiatric nurse practitioners, physician assistants, and primary care providers should include updated guidance on the indications, administration, and communication strategies around LAIs. Health systems should reduce administrative and logistical barriers that limit access to LAIs, including prior authorizations and inadequate reimbursement. Community pharmacists should be increasingly utilized to deliver LAI injections close too patients’ homes and in non-stigmatizing settings. Additionally, public education campaigns can also play a role in normalizing LAI use by dispelling myths and rectifying misconceptions, and promoting early intervention for people with schizophrenia and other psychotic disorders more broadly.

As the field continues to refine early intervention strategies, integrating LAIs alongside psychosocial supports offers a meaningful and proactive pathway to optimizing recovery opportunities for patients with schizophrenia, especially early in their illness. It is time to align clinical practice with the accumulated evidence and challenge outdated assumptions, reframing LAIs as proactive, evidence-based and recovery-oriented tools that are a cornerstone of schizophrenia care from the very first episode, which can help facilitate patients’ improved goal attainment.

## CNS SPECTRUMS

### CME Review Article

**Long-Acting Injectable (LAIs) Antipsychotics for Patients with First-Episode and Early-Phase Schizophrenia: Still Not Considered Often Enough**

*This CME activity is provided by HMP Education and Neuroscience Education Institute (NEI).*

### CME/CE Information

**Target Audience:** This activity has been developed for the healthcare team or individual prescriber specializing in mental health. All other healthcare team members interested in psychopharmacology are welcome for advanced study.

**Learning Objectives:** After completing this educational activity, you should be better able to:

- Delineate the opportunities and challenges of patients with first-episode and early-phase schizophrenia
- Identify the specific use case of LAIs in patients with first-episode and early-phase schizophrenia
- Appreciate the randomized controlled and observational data of LAIs in patients with first-episode and early-phase schizophrenia, as well as data supporting newer indications for bipolar I disorder

**Activity Overview:** This activity is best supported via a computer or device with current versions of the following browsers: Mozilla Firefox, Google Chrome, or Safari. A PDF reader is required for print publications. A post-test score of 70% or higher is required to receive CME/CE credit.

**Estimated Time to Complete:** 1 hour.

Continuing Education credit will be available for three (3) years from the publication date of the associated article. Please visit https://nei.global/cnsspectrums2025 for additional information and to access the CE activity.

**NEI maintains a record of participation for six (6) years.*

### Instructions for Optional Posttest and CME Credit

1. Read the article
2. Successfully complete the posttest at https://nei.global/CNS/LAI-02
3. Print your certificate

Questions? Email customerservice@neiglobal.com.

**Credit Designations:** The following are being offered for this activity:

- **Physician: ACCME *AMA PRA Category 1 Credits*™**
  - HMP Education designates this enduring material for a maximum of 1.00 *AMA PRA Category 1 Credit*™. Physicians should claim only the credit commensurate with the extent of their participation in the activity.
- **Nurse: ANCC contact hours.**
  - This continuing nursing education activity awards 1.00 contact hour. Provider approved by the California Board of Registered Nursing, Provider #18006 for 1.00 contact hour.
- **Nurse Practitioner: ACCME *AMA PRA Category 1 Credit*™**
  - American Academy of Nurse Practitioners National Certification Program accepts *AMA PRA Category 1 Credits*™ from organizations accredited by the ACCME.
  - The content in this activity pertaining to pharmacology is worth 1.00 continuing education hour of pharmacotherapeutics.
- **Pharmacy: ACPE application-based contact hours.**
  - This internet enduring, knowledge-based activity has been approved for a maximum of 1.00 contact hour (.10 CEU).
  - *The official record of credit will be in the CPE Monitor system. Following ACPE Policy, NEI and HMP Education must transmit your claim to CPE Monitor within 60 days from the date you complete this CPE activity and are unable to report your claimed credit after this 60-day period. Ensure your profile includes your DOB and NABP ID.*
- **Physician Associate/Assistant: AAPA Category 1 CME credits.**
  - HMP Education has been authorized by the American Academy of PAs (AAPA) to award AAPA Category 1 CME credits for activities planned in accordance with the AAPA CME Criteria. This internet enduring activity is designated for 1.00 AAPA Category 1 credit. Approval is valid until February 11, 2028. PAs should only claim credit commensurate with the extent of their participation.
- **Psychology: APA CE credits.**
  - Continuing Education (CE) credits for psychologists are provided through the co-sponsorship of the American Psychological Association (APA) Office of Continuing Education in Psychology (CEP). The APA CEP Office maintains responsibility for the content of the programs. This activity awards 1.00 CE Credit.
- **Social Work: ASWB-ACE CE credits.**
  - As a Jointly Accredited Organization, HMP Education is approved to offer social work continuing education by the Association of Social Work Boards (ASWB) Approved Continuing Education (ACE) program. Organizations, not individual courses, are approved under this program. Regulatory boards are the final authority on courses accepted for continuing education credit. Social workers completing this internet enduring course receive 1.00 general continuing education credit.
- **Non-Physician Member of the Healthcare Team: Certificate of Participation.**
  - HMP Education awards hours of participation (consistent with the designated number of *AMA PRA Category 1 Credit*™) to a participant who successfully completes this educational activity.

**Peer Review:** The content was peer-reviewed by an MD — to ensure the scientific accuracy and medical relevance of information presented and its independence from commercial bias. NEI and HMP Education take responsibility for the content, quality, and scientific integrity of this CME/CE activity.

**Disclosures:** All individuals in a position to influence or control content are required to disclose any relevant financial relationships. Any relevant financial relationships were mitigated prior to the activity being planned, developed, or presented.
***Faculty Author / Presenter***

**Christopher U. Correll, MD**
*Professor of Psychiatry and Molecular Medicine, Zucker School of Medicine at Hofstra/Northwell, NY*
*Professor of Child and Adolescent Psychiatry at Charité University Medicine, Berlin, Germany* Consultant/Advisor: AbbVie, Alkermes, Allergan, Angelini, Aristo, Autobahn, Boehringer-Ingelheim, Bristol-Meyers Squibb, Cardio Diagnostics, Cerevel, CNX Therapeutics, Compass Pathways, Darnitsa, Delpor, Denovo, Draig, Eli Lilly, Eumentis Therapeutics, Gedeon Richter, Hikma, Holmusk, IntraCellular Therapies, Jamjoom Pharma, Janssen/J&J, Karuna, LB Pharma, Lundbeck, MedInCell, MedLink, Merck, Mindpax, Mitsubishi Tanabe Pharma, Maplight, Mylan, Neumora Therapeutics, Neuraxpharm, Neurocrine, Neurelis, Newron, Noven, Novo Nordisk, Otsuka, PPD Biotech, Recordati, Relmada, Response Pharmaceuticals, Reviva, Rovi, Saladax, Sanofi, Seqirus, Servier, Sumitomo Pharma America, Sunovion, Sun Pharma, Supernus, Tabuk, Takeda, Teva, Terran, Tolmar, Vertex, Viatris, Xenon Pharmaceuticals Speakers Bureau: Janssen, Lundbeck, Otsuka Board Member: Compass Pathways, IntraCellular Therapies, Relmada, Reviva, Rovi Grant/Research: Boehringer-Ingelheim, Janssen, Takeda Stock Options: Cardio Diagnostics, Kuleon Biosciences, LB Pharma, Medlink, Mindpax, Quantic, Terran The remaining Planning Committee members, Content Editors, Peer Reviewer, and NEI planners/staff have no financial relationships to disclose. NEI and HMP Education planners and staff include Meghan Grady, Caroline O’Brien, MS, Ali Holladay, Moriah Carswell, Andrea Zimmerman, EdD, CHCP, and Brielle Calleo

**Disclosure of Off-Label Use:** This educational activity may include discussion of unlabeled and/or investigational uses of agents that are not currently labeled for such use by the FDA. Please consult the product prescribing information for full disclosure of labeled uses.

**Cultural Linguistic Competency and Implicit Bias:** A variety of resources addressing cultural and linguistic competencies and strategies for understanding and reducing implicit bias can be found in this handout—download me.

### Accessibility Statement

For questions regarding this educational activity, or to cancel your account, please email customerservice@neiglobal.com.

**Support:** This activity is supported by an unrestricted educational grant from Alkermes, Inc., Teva Pharmaceuticals, Johnson & Johnson Innovative Medicine and Otsuka America Pharmaceutical, Inc.
