# Long-acting injectable antipsychotics for patients with first-episode and early-phase schizophrenia: still not considered often enough – ERRATUM

**DOI:** 10.1017/S1092852925100655

**Published:** 2025-11-04

**Authors:** Christoph U. Correll

Cambridge University Press apologises for an error in the originally published article. The CME/CE Information was not originally included in the article PDF. This has now been updated.
